# Longitudinal Assessment of Abnormal Cortical Folding in Fetuses and Neonates With Isolated Non‐Severe Ventriculomegaly

**DOI:** 10.1002/brb3.70255

**Published:** 2025-01-20

**Authors:** Andrea Urru, Oualid Benkarim, Gerard Martí‐Juan, Nadine Hahner, Gemma Piella, Elisenda Eixarch, Miguel A. González Ballester

**Affiliations:** ^1^ BCN MedTech, Department of Information and Communication Technologies Universitat Pompeu Fabra Barcelona Spain; ^2^ McConnell Brain Imaging Centre Montreal Neurological Institute and Hospital, McGill University Montreal Quebec Canada; ^3^ BCNatal Fetal Medicine Research Center (Hospital Clínic and Hospital Sant Joan de Déu) University of Barcelona Barcelona Spain; ^4^ Department of Surgery and Surgical Specializations, Faculty of Medicine and Health Sciences University of Barcelona Barcelona Spain; ^5^ Institut d'Investigacions Biomèdiques August Pi i Sunyer (IDIBAPS) and Centre for Biomedical Research on Rare Diseases (CIBERER) Barcelona Spain; ^6^ ICREA Barcelona Spain

**Keywords:** atlas‐based segmentation | brain | fetal | longitudinal analysis | mixed‐effects model | MRI | neonatal | ventriculomegaly

## Abstract

**Purpose:**

The impact of ventriculomegaly (VM) on cortical development and brain functionality has been extensively explored in existing literature. VM has been associated with higher risks of attention‐deficit and hyperactivity disorders, as well as cognitive, language, and behavior deficits. Some studies have also shown a relationship between VM and cortical overgrowth, along with reduced cortical folding, both in fetuses and neonates. However, there is a lack of longitudinal studies that study this relationship from fetuses to neonates.

**Method:**

We used a longitudinal dataset of 30 subjects (15 healthy controls and 15 subjects diagnosed with isolated non‐severe VM (INSVM)) with structural MRI acquired in and ex utero for each subject. We focused on the impact of fetal INSVM on cortical development from a longitudinal perspective, from the fetal to the neonatal stage. Particularly, we examined the relationship between ventricular enlargement and both volumetric features and a multifaceted set of cortical folding measures, including local gyrification, sulcal depth, curvature, and cortical thickness.

**Findings:**

Our results show significant effects of isolated non‐severe VM (INSVM) compared to healthy controls, with reduced cortical thickness in specific brain regions such as the occipital, parietal, and frontal lobes.

**Conclusion:**

These findings align with existing literature, confirming the presence of alterations in cortical growth and folding in subjects with isolated non‐severe VM (INSVM) from the fetal to neonatal stage compared to controls.

## Graphical abstract

This study investigates the longitudinal impact of isolated non‐severe ventriculomegaly (INSVM) on cortical development from fetal to neonatal stages using MRI data from 30 subjects (15 with VM and 15 healthy controls). The results indicate that VM subjects exhibit larger cortical volume, reduced cortical thickness and altered local gyrification over time, particularly in the occipital, parietal, and frontal lobes, confirming cortical overgrowth and delayed cortical folding observed in cross‐sectional studies.

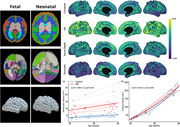

## 1 Introduction

Ventriculomegaly (VM) is a pathological condition defined by an enlargement of the brain's ventricular system (Scott et al. 2013; Kyriakopoulou et al. 2014; Benkarim et al. 2018; Benkarim et al. 2020; Benkarim et al. 2017). It is one of the most common brain anomalies diagnosed in fetuses, with an incidence of around 1% of pregnancies (Salomon et al. 2007; Huisman et al. 2012). VM can affect one or both ventricles, and it is diagnosed by a lateral ventricle atrial diameter exceeding 10 mm at any stage of gestation from the 14th week onwards. This condition has been associated with various neurological disorders in adults and children, including schizophrenia, autism, and attention deficit (Landrieu et al. 1998; Sallet et al. 2003; Nordahl et al. 2007; Jou et al. 2005; Wolosin et al. 2009). Furthermore, several studies have highlighted links between VM and anomalies in cortical development, such as cortical overgrowth in fetuses (Kyriakopoulou et al. 2014; Benkarim et al. 2018), also reporting associations of VM with cortical volume and the degree of cortical folding, and white and grey matter alterations in neonates (Lockwood Estrin et al. 2016).

When VM is diagnosed in utero, postnatal prognosis depends on the presence of other abnormalities and the severity and progression of ventricular dilation, all associated with poorer outcomes. In this work, we focus on isolated non‐severe VM (INSVM), which is diagnosed by a ventricular atrial diameter between 10 and 15 mm and in the absence of other abnormalities. Despite the majority of INSVM‐diagnosed fetuses not showing long‐term neurodevelopmental deficits (Griffiths et al. 2010; Melchiorre et al. 2009) compared to more severe presentations (Ali et al. 2024), some may experience unfavorable outcomes (Sadan et al. 2007; Leitner et al. 2009; Gómez‐Arriaga et al. 2012). Identifying the factors that potentially influence these postnatal outcomes is crucial for informed clinical counseling and decision‐making.

Understanding how VM affects brain growth involves examining brain structures, particularly the gyri and sulci and their development. Gyri are convex regions surrounded by concave sulci, which deepen into fissures dividing brain lobes and hemispheres. This interplay allows for a larger cortical surface area, supporting more neuronal connections without proportionally enlarging the brain. Although not fully understood (Toro and Burnod 2005; Fernández et al. 2016), these convolutional patterns are essential for cortical specialization. Primary sulci, like the Sylvian fissure, develop consistently across individuals (Chi et al. 1977; Armstrong et al. 1995; Griffiths et al. 2010), while secondary and tertiary sulci form more irregularly, with their development continuing into the neonatal stage (Voorhies et al. 2021).

Neurodevelopmental delays and cognitive impairment in neonates have been associated with alterations in cortical development. For instance, studies employing magnetic resonance imaging (MRI) found compelling links between cortical changes and a range of developmental issues. Limperopoulos et al. (2014) discovered correlations between autism signs and cortical volume alterations in preterm infants. Dean et al. (2013) showed that prenatal cerebral ischemia can disrupt cortical neuron differentiation. Additionally, reductions in cortical volumes and delayed gyrification have been observed in neonates with severe congenital heart disease (Claessens et al. 2016). Notably, Neumane et al. (2022) identified differences in sensorimotor white matter between preterm and full‐term infants, with effects escalating with earlier birth. Several works also leveraged 3D MRI to model and analyze fetal brain development in both healthy (Habas et al. 2010; Kuklisova‐Murgasova et al. 2011; Serag et al. 2012) and non‐healthy subjects (Payette et al. 2021; Payette et al. 2020; de Dumast et al. 2020). While the former focused on establishing normative models of brain development, the latter identified deviations from these norms as potential biomarkers for diseases. These studies highlight the role of MRI in investigating the relationship between cortical alterations and neurodevelopmental issues during fetal and neonatal development.

For VM, MRI is indicated for both in‐ and ex utero diagnosis (Rutherford 2002). Several studies have explored the connection between ventricular enlargement and cortical development in VM (Scott et al. 2013; Kyriakopoulou et al. 2014; Benkarim et al. 2018; Benkarim et al. 2020; Gilmore et al. 2008; Lyall et al. 2012; Lockwood Estrin et al. 2016). Nonetheless, these studies were either cross‐sectional, composed of only fetuses (Scott et al. 2013; Kyriakopoulou et al. 2014; Benkarim et al. 2018; Benkarim et al. 2020), or neonates (Gilmore et al. 2008; Lyall et al. 2012), or longitudinal but only on neonates (Lockwood Estrin et al. 2016), which prevents us from having a clear picture of the longitudinal changes in cortical development of fetuses diagnosed with VM.

In this paper, we used a longitudinal dataset comprised of 30 subjects, split evenly between 15 healthy controls and 15 individuals diagnosed with INSVM. Our objective was to study the relationship, both cross‐sectionally and longitudinally, of ventricular enlargement with both volumetric features and a multifaceted set of cortical folding measures, namely local gyrification, sulcal depth, curvature, and cortical thickness. Both global and local differences between subjects with INSVM and healthy controls were analyzed. Structural MRI scans were acquired both in and ex utero for each subject, marking, to our knowledge, the first longitudinal study to investigate VM's impact on cortical development from the fetal to the neonatal stage.

## 2 Materials and Methods

### 2.1 Dataset

The dataset used in this study consisted of longitudinal MRI acquisitions from a subset of participants in a research project on INSVM conducted at Hospital Clínic in Barcelona, Spain. The study protocol received approval from the Ethics Committee of Hospital Clínic (HCB/2014/0484), and all participants provided written informed consent.

The dataset included MRI scans of 30 subjects from a larger prospective cohort of 81 subjects (Hahner et al. 2019), divided in 15 healthy controls and 15 subjects with INSVM. Diagnosis was based on the ventricular diameter by ultrasound, following international guidelines (Malinger et al. 2020). Isolated non‐severe VM was defined as having an atrial width above 10.0 at sonographic examination. Atrial width was assessed with the atrial being the region of the lateral ventricle at the junction of the posterior and inferior horns. All fetuses were from singleton pregnancies and met the inclusion criteria with normal karyotype and no perinatal infections or malformations with risk of abnormal neurodevelopment. No subjects with INSVM required any surgical procedures. All acquired MRI scans were visually inspected for apparent or aberrant artifacts and brain anomalies, either at acquisition time or during the preprocessing process, excluding a total of 51 scans from the original dataset of 81.

The age range at the time of scan was 26.2 to 33.7 weeks post‐menstrual age (PMA) for fetal acquisitions, while neonatal images had an age range between 41.29 to 47.57 weeks PMA. The whole dataset was thus composed of a total of 60 images, two for each subject. Table 1 shows the demographics of the complete dataset. Additional demographic information on the cohort regarding ethnicity, socioeconomic status, and education can be found in the Supporting Information S1.

**TABLE 1:** Demographics.

		Control	INSVM	*P*‐value
N		15	15	
Sex (M/F)		9/6	13/2	0.08
ΔTime (weeks)		14.06±3.9	14.8±1.9	0.49
Age (weeks)	Fetal	29.6±3.1	28.7±2.3	0.17
	Neonatal	43.6±1.4	43.6±1.7	0.46
VV (cm^3^)	Fetal	3.74±1.16	9.87±4.04	<0.001
	Neonatal	5.18±1.00	12.86±5.71	<0.001

*Note*: The table reports the number of participants in each group (i.e., healthy controls and subjects with INSVM), the proportion of males and females (M/F) in each group, the mean (± standard deviation) ∆time between the two scans in weeks, mean (± standard deviation) age and ventricular volume (VV) for each group and timepoint (i.e., fetal and neonatal). Group differences were assessed using a *t*‐test for continuous variables (i.e., age, ventricular volume) and a chi‐squared test for binary variables (i.e., sex).

### 2.2 MRI Acquisition Fetuses

For in utero acquisitions, T2‐weighted MRI was performed on a 1.5‐T scanner (SIEMENS MAGNETOM Aera syngo MR D13; Munich, Germany) with an 8‐channel body coil. All images were acquired without sedation and following the American College of Radiology guidelines for pregnancy and lactation. Half Fourier acquisition single‐ shot turbo spin echo (HASTE) sequences were used with the following parameters: repetition time (TR) = 1500 ms, echo time (TE) = 82 ms, number of averaging = 1, slice thickness = 2.5 mm, field of view (FOV) = 280 × 280 mm, and voxel size = 0.5 × 0.5 × 2.5 mm. For each subject, multiple orthogonal acquisitions were performed: 4 axial, 2 coronal, and 2 sagittal stacks. Brain location in each 2D slice was carried out in an automatic manner using the approach proposed by Salehi et al. (2018), followed by high‐resolution 3D volume reconstruction using the method presented in Ebner et al. (2018, 2019).

#### 2.2.1 Neonates

Neonatal images were acquired during natural sleep using a TIM TRIO 3.0T whole‐ body MR scanner (Siemens, Germany). T2‐weighted images were obtained with the following parameters: 2‐mm slice thickness with a 2 mm inter‐slice gap, an in‐plane acquisition matrix of 256 × 256, and an FOV = 160 × 241 mm, which resulted in a voxel dimension of 0.625 × 0.625 × 2 mm, TR = 5980 ms, and TE = 91 ms. Supporting Information S2 shows axial views at the atrial level of fetal and neonatal subjects’ brain comparing healthy controls and subjects with INSVM.

### 2.3 Processing Pipeline

**FIGURE 1:**
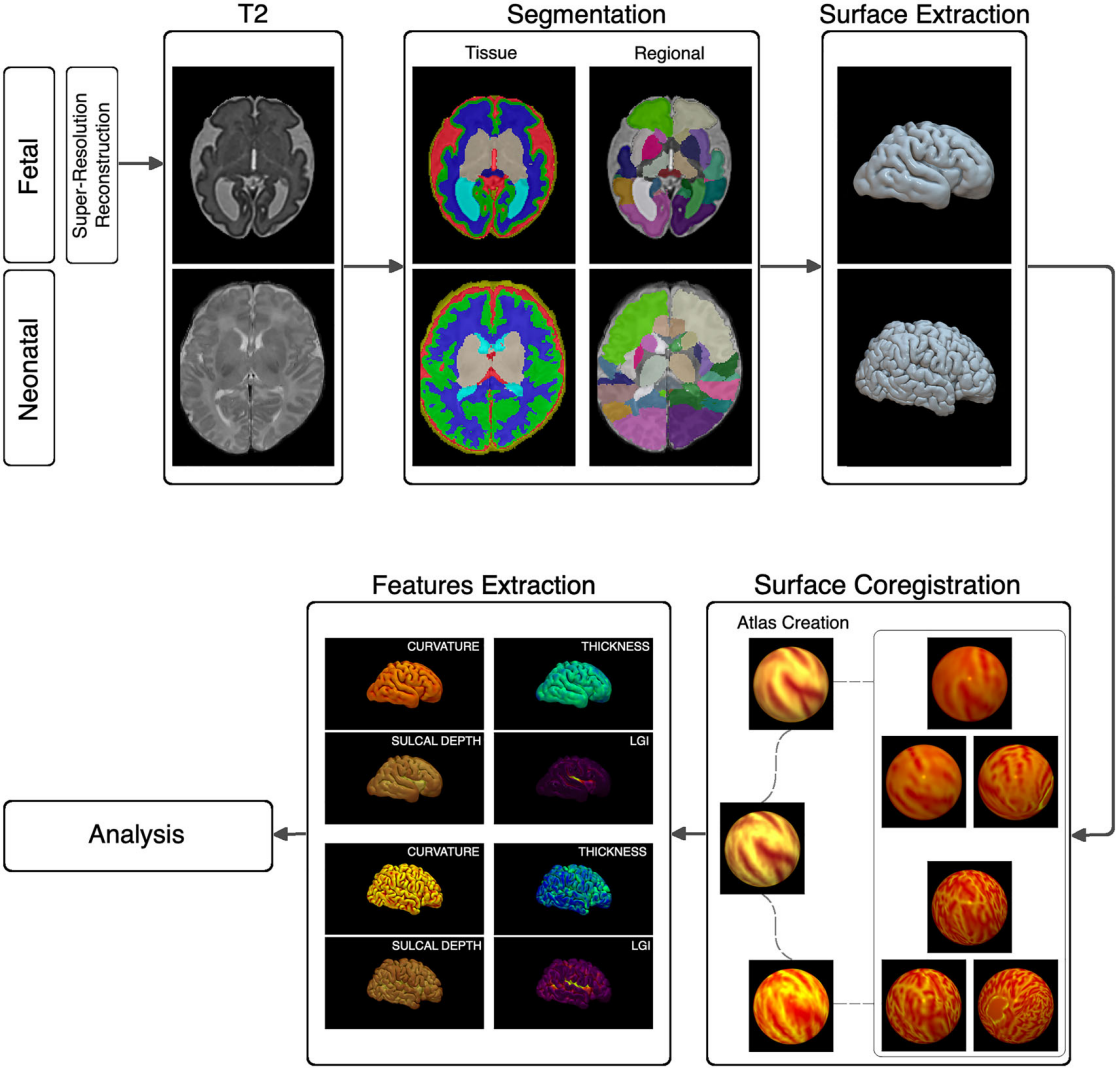
Processing pipeline. After performing a high‐resolution reconstruction for fetal images, we generated tissue and regional segmentations. Next, inner and outer cortical surfaces were generated, and cortical features (i.e., mean curvature, sulcal depth, thickness, and local gyrification index) were computed. Spherical Demons was then used to establish vertex wise correspondences between individual surfaces within the same timepoint (i.e., between fetuses and between neonates), followed by atlas generation for fetuses and neonates, and the alignment between these atlases established vertex‐wise correspondence between fetal and neonatal surfaces.

Both fetal and neonatal brain images were segmented using the automatic segmentation pipeline described in Urru et al. (2023). Briefly, the pipeline uses an atlas‐based label fusion segmentation approach based on atlases with a similar age to that of the target image. The pipeline produced tissue‐level and regional segmentations for each subject. Once the segmentations of the main tissues were obtained, inner and outer cortical surfaces were extracted. The following cortical features were computed for each subject: mean curvature, cortical thickness, sulcal depth, and local gyrification index (LGI). Figure 1 shows the entire processing pipeline, extending from image acquisition to feature extraction.

#### 2.3.1 Segmentation and Surface Extraction

Segmentation proceeded with a brain‐extraction step, implemented using the brain extraction tool (BET) (Smith 2002) to remove the remaining extra‐brain tissue (e.g., parts of the skull) in both fetuses and neonates. Once the brain was extracted, it was rigidly registered to the corresponding template and intensity inhomogeneity corrected.

The segmentation was then executed using an expectation‐maximization algorithm. Each subject was initially registered, using image intensity, to a temporal template, and a first tissue segmentation estimation was computed accordingly. In a subsequent step, a three‐channel registration, utilizing the T2‐weighted image, grey matter, and ventricle probability maps, was performed against a multisubject atlas. This registration facilitated the computation of a regional parcellation and further refinement of the tissue segmentation derived in the previous step.

Next, white‐matter and pial surfaces were extracted. For each subject, a triangulated surface mesh was fit onto the computed white‐matter segmentation boundary. The pial surface was obtained by deforming the white‐matter mesh towards the boundary between cortical gray matter (cGM) and cerebrospinal fluid (CSF), looking for the closest cGM/CSF image edge outside the white‐matter mesh, and adding a regularization term to smoothen the resulting mesh. A comprehensive description and applications of the processing pipeline can be found in Urru et al. (2023).

#### 2.3.2 Atlas Creation

The cortical surface meshes extracted from the previous step were not in vertex‐wise correspondence. To standardize the topology and establish correspondence between vertices, our cortical surfaces were registered to a common reference frame using Spherical Demons (Yeo et al. 2010). This algorithm performs the registration of two surfaces based on their spherical projections with geometric features at each vertex (e.g., mean curvature). In this case, given the considerable differences between fetal and neonatal cortices, Spherical Demons was performed separately for each hemisphere and timepoint. This procedure resulted in a total of four spherical atlases, each one representing a hemisphere and timepoint. Subsequently, each mesh was regularized based on the atlas size and topology by means of the iterative closest point (ICP) algorithm (Besl and McKay 1992). These steps brought the spherical projections in correspondence, for each timepoint, and the cortical meshes were remeshed accordingly.

To perform a longitudinal vertex‐wise analysis, it is crucial to register both fetal and neonatal surfaces to a shared reference frame. Although the neonatal cortex is substantially more convoluted than its fetal counterpart, prominent cortical landmarks such as primary sulci and gyri, formed as early as the 14th gestational week, can be identified at both time points (e.g., central and lateral sulci). These landmarks are likely to drive the coregistration process between the fetal and neonatal atlases. Following this, the Spherical Demons algorithm was applied again to the two sets of previously computed atlases. As a result, a pair of atlases was obtained (one for each hemisphere), which brought all the subjects into correspondence across both time points.

#### 2.3.3 Characterization of Cortical Folding

Upon extraction of the cortical surfaces, four distinct geometric features were computed to characterize cortical folding. These included mean curvature, local gyrification index, sulcal depth, and thickness.

2.3.3.1 | Mean Curvature. Mean curvature is estimated from the average of the principal curvatures of the inner surface. The principal curvatures represent the minimum and maximum bending of a regular surface at each given point (King et al. 2016). As the cortex expands and becomes more convoluted, this descriptor generally increases. In contrast, at an earlier developmental stage, the simpler shape of the cortical plate corresponds to a lower mean curvature.

2.3.3.2 | Local Gyrification Index. This descriptor is calculated as the ratio between an area taken on the brain's cortical surface and the area covered by the same points on the inflated surface. The inflation process moves outward the points in the sulci and brings in the points on the gyral crowns, preserving distance between neighboring vertices until a certain level of smoothness is reached (Lyu et al. 2018). On the average fetal brain, it is observed that the LGI approaches zero where the surface is flat, while it reaches its highest values in the deepest parts of the main sulci.

2.3.3.3 | Sulcal Depth. Sulcal depth represents the distance of a vertex on the convoluted cortical surface from the corresponding vertex on the convex hull obtained by inflating the cortical surface (Yun et al. 2013). Being associated with the degree of gyrification of the cortex, sulcal depth generally increases with brain development.

2.3.3.4 | Thickness. Cortical thickness is defined as the average distance between two measures: (1) the Euclidean distance from the inner surface to the closest vertex on the outer (i.e., pial) surface and (2) the Euclidean distance from the pial surface to the closest vertex on the inner surface. Cortical thickness is at its maximum during early development, and then gradually diminishes as the cortex thins to accommodate bending as it grows (Sowell et al. 2004; Brown et al. 2012; Amlien et al. 2016).

#### 2.3.4 Global Analysis

In our initial analysis, we investigated global group differences based on the volumes of the main tissues as well as on cortex‐wide folding features. Ventricles, cortical grey matter, white matter, and supratentorial volumes were all computed based on the previously generated segmentations. The features were corrected for age and sex effects, and the distributions of the two groups were log‐transformed to normalize them. A Student's t‐test was then used to assess the differences between the groups for each cortical folding feature and at each timepoint.

#### 2.3.5 Volumetric Analysis

Following this, we proceeded to analyze lobular volumes, analyzing between‐group differences at each time point as well as longitudinally. For this analysis we used regional parcellations, which divided the cortex into six different regions: the cingulate gyrus, insula, frontal, occipital, parietal, and temporal areas. Group‐level growth trajectories were fitted for each of these regions to analyze the differences. Furthermore, we investigated the percentage mean difference for each time point separately after correcting for the effects of age and sex.

Finally, we also examined local and global volume differences from a longitudinal perspective, using a mixed‐effects model (Raudenbush and Bryk 2002) to predict the volume of each of the aforementioned cortical areas. A mixed‐effects model combines fixed and random effects, and it allows to model independent multiple acquisitions of the same subjects through time. In our case, we acquired two images for each subject, at fetal and neonatal stages. We tested the effect of diagnosis on ventricular volume, cortical volume, and lobular volumes, with age and sex as fixed‐effect covariates and the multiple acquisitions as a random effect:

(1) $$V_{\mathrm{lobe}}=\beta_{0}+\beta_{1}\mathrm{GA}+\beta_{2}S+\beta_{3}\mathrm{DG}+\alpha \mathrm{ID}$$

where *V* represents the predicted volume, GA is the age in weeks, S denotes the sex of the subjects (0 for female, 1 for male), DG stands for diagnosis (0 for control, 1 for INSVM), while ID is a unique identifier for each subject.

#### 2.3.6 Morphometric Analysis

We conducted a vertex‐wise morphometric analysis to assess the relationship between ventricular enlargement and cortical folding, characterized using the four aforementioned descriptors. The analysis was carried out using a mixed‐effects model to incorporate the two acquisitions (fetal and neonatal) for each subject and to predict the cortical folding features at each vertex. Specifically, we used a more complex model here, introducing interactions between covariates to establish the effect of diagnosis (i.e., the presence of abnormal ventricular development) on cortical folding:

(2) $$F_{i}=\beta_{0}+\beta_{1}\mathrm{GA}+\beta_{2}S+\beta_{3}\mathrm{DG}+\beta_{4}\mathrm{GA}\times \mathrm{VV}+\alpha \mathrm{ID}$$

where F*_i_* represents the modeled cortical feature, GA is the age in weeks, *S* the sex of the subjects (0 for female, 1 for male), DG the diagnosis (0 for control, 1 for INSVM), VV represents ventricular volume, and ID is a unique identifier for each subject. We also included interactions between age and ventricular volume to capture ”normal” cortical development, and, in the case of curvature and sulcal depth, we added an extra quadratic term for age. We applied family‐wise error correction with random field theory (RFT) to account for spatial correlation (Hayasaka et al. 2004), with a cluster threshold of 0.05, and further removed clusters with 50 or fewer vertices, ensuring the robustness of detected effects.

We also assessed the relationship between the map of group‐wise differences in local gyrification index and those of the remaining cortical folding descriptors (i.e., group‐wise differences in mean curvature, sulcal depth, and cortical thickness) using Spearman's correlation. For this analysis, we controlled for spatial autocorrelations using nonparametric permutation tests (i.e., spin tests) (Alexander‐Bloch et al. 2018).

## 3 Results

### 3.1 Global Analysis

**FIGURE 2:**
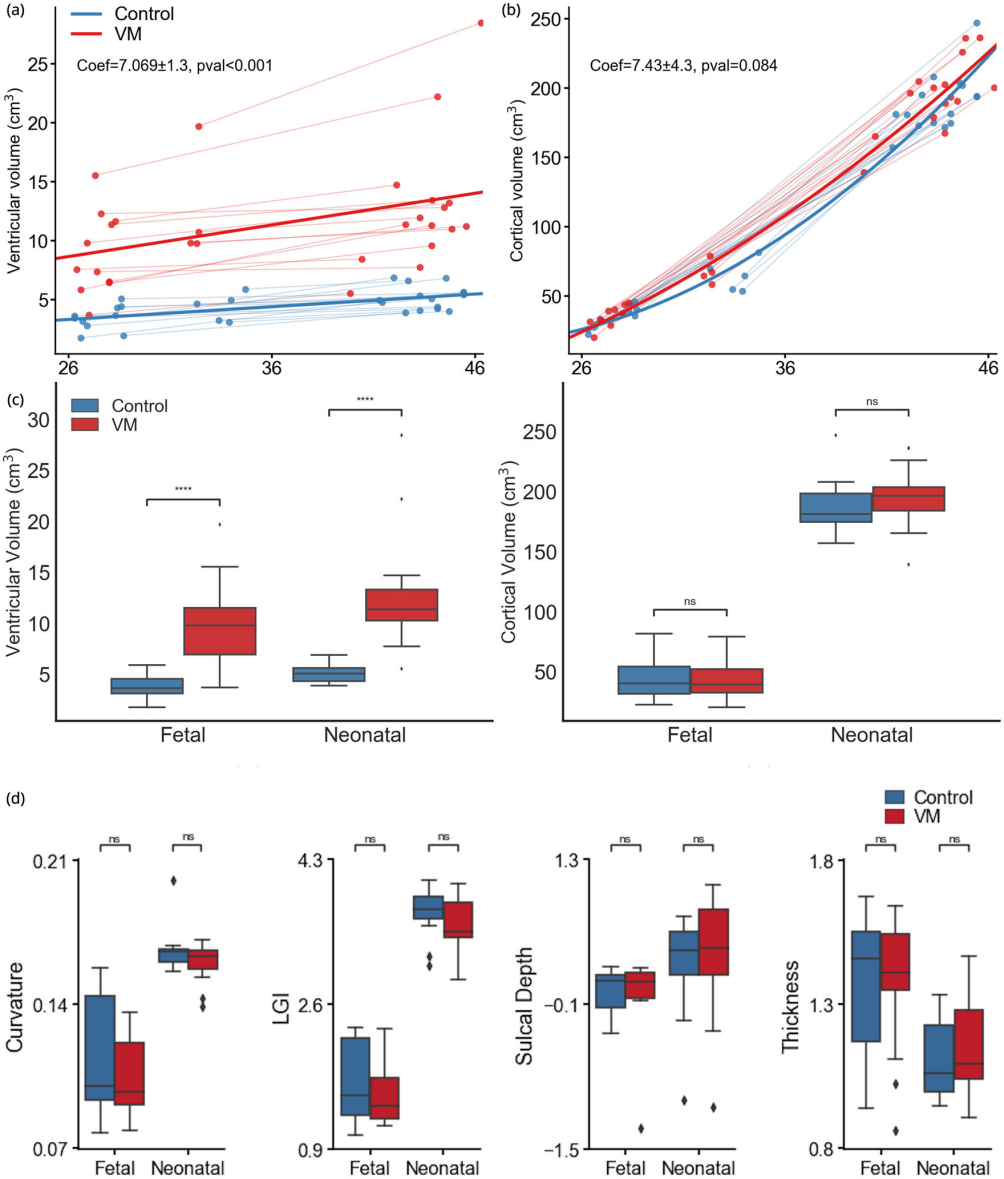
Global differences in volumetric and morphometric developmental trajectories. (a) Total ventricular volume (VV) growth trajectories for healthy controls and subjects with INSVM (from fetal to neonatal stage). Coefficient effect, standard deviation, and *p*‐value of the effect of the diagnosis over VV on a mixed‐effect model, correcting for age and sex. (b) Cortical volume growth trajectories for healthy controls and subjects with INSVM. Group‐level and individual trajectories are displayed using solid and dashed lines, respectively. Coefficient effect, standard deviation, and *p*‐value of the effect of the diagnosis over cortical volume on a mixed‐effect model, correcting for age and sex. (c) Group‐ wise distribution of ventricular volume and cortical volume at both fetal and neonatal stages. Boxes denote the interquartile range (IQR) between the first and third quartiles, and the line inside denotes the median. Whiskers extend to points that lie within 1.5 IQRs of the lower and upper quartiles, and the black diamonds denote outliers. Comparison between groups was done using a generalized linear model, including gestational age and sex as covariates. (d) Group‐wise distributions of mean curvature, cortical thickness, local gyrification index (LGI), and sulcal depth at both fetal and neonatal stages. Comparison between groups done using a generalized linear model, including gestational age and sex as covariates. * *p* < 0.05, **p < 0.01, and ***p < 0.001. NS, non‐significant.

Our initial analysis focused on global differences in brain development, specifically ventricular and cortical volumes, and the previously described morphometric features averaged throughout the entire cortex. These variables were used to quantify differences between controls and subjects with INSVM at the two investigated timepoints. Figure 2 shows the difference between controls and subjects with INSVM in terms of ventricular and cortical volume, respectively. Both single‐subject and group‐level growth trajectories are included.

We identified significant differences both in volumetric and morphometric features between the groups. Ventricular volumes showed strong significant differences between controls and INSVM that remained consistent over time (7.069 ± 1.3 cm^3^, *p* < 0.001). Differences were also found in terms of cortical volume, with subjects with INSVM exhibiting a steeper growth in cortical volume from early to late‐onset fetuses in comparison to controls, but with less differences and too much variation across subjects to be a significant effect (7.43 ± 4.3 cm^3^, *p* = 0.084).

Turning to global morphometric features, we detected no significant differences in any of the geometric features, either for the fetal or neonatal stages, after correcting for gestational age and sex. For fetuses, all values are lower in INSVM compared to controls. For neonates we observed a reduction of both curvature (−0.03, with *t* = −1.54, *p* = 0.12) and local gyrification index (−0.038, with *t* = −1.08, *p* = 0.27) in INSVM. Higher values of cortical thickness in subjects with INSVM at the neonatal stage were also observed (increase of 0.02, with t = 0.55, *p* = 0.57).

### 3.2 Volumetric Analysis

**FIGURE 3:**
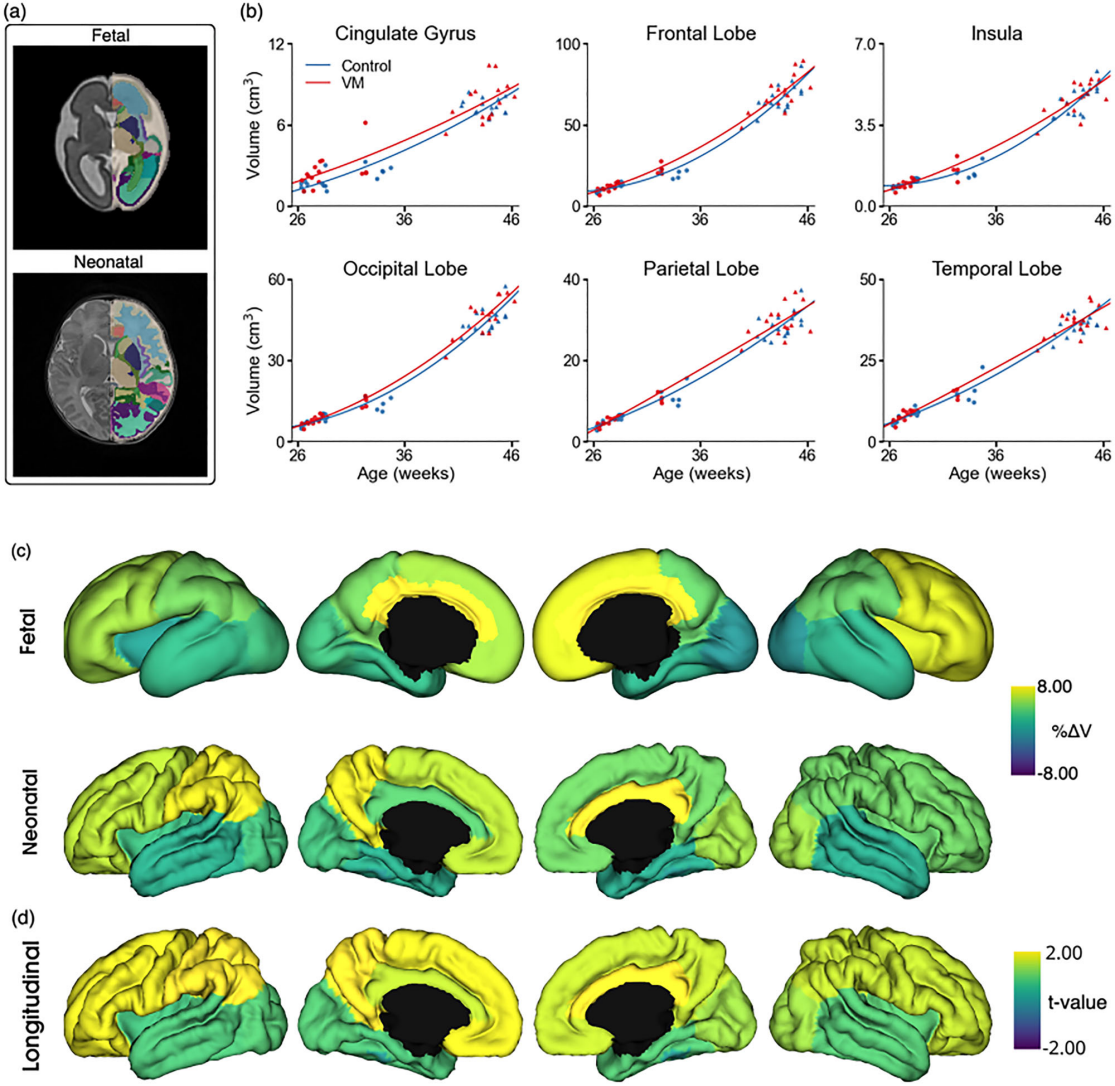
Volumetric analysis. (a) Regional brain parcellations of the same subject (diagnosed with INSVM) at both fetal and neonatal stages. (b) Growth trajectories of lobular volumes (in cm) plotted against gestational age (in weeks) for controls (blue) and INSVM subjects (red) across six brain regions: cingulate gyrus, frontal lobe, insula, occipital lobe, parietal lobe, and temporal lobe. (c) Percentage volume differences (%∆*V*) between INSVM and control groups mapped onto the fetal and neonatal average brains. Yellow regions indicate larger volumes in INSVM subjects relative to controls, while blue regions indicate smaller volumes. (d) *T*‐statistics (*t*‐values) from a mixed‐effects model, adjusted for age and sex, showing the statistical significance of volume differences between the INSVM and control groups across brain regions. Yellow regions indicate larger volumes in INSVM subjects relative to controls, while blue regions indicate smaller volumes.

Figure 3 shows the volumetric development disparities across the various lobes of the cortex. We computed the mean percentage differences between the cohorts for each timepoint after adjusting for age and sex effects. Then, in the longitudinal analysis, we used a mixed‐effects model to predict each lobular volume and find the regions in which volume significantly differed between subjects with INSVM and controls.

Results show a consistent pattern of volumetric growth in the insula, frontal, occipital, and parietal lobes, where a steeper growth trajectory was observed in subjects with INSVM from early to late‐onset compared to controls. Significant differences were observed in the right cingulate, with an average increase of 0.292 cm^3^ in cortical volume (*t* = 1.96, *p* = 0.049) in INSVM with respect to controls, and in the left occipital, showing an average increase of 1.12 cm^3^ in volume (*t* = 2.01, *p* = 0.044). The full results of the mixed‐effects models can be found in Supporting Information S3.

We additionally performed analyses using generalized linear models separately for fetal and neonatal volumes, as well as for fetal to neonatal change, correcting for age and sex. No significant differences were observed, except for a larger volume in the right cingulate gyrus in fetal subjects with INSVM compared to controls (0.34 ± 0.15 cm^3^), which is in agreement with the mixed models results. These results suggest that the primary differences in cortical volume we detected before manifest during the fetal stage. A detailed table of results is provided in Supporting Information S4.

### 3.3 Morphometric Analysis

**FIGURE 4:**
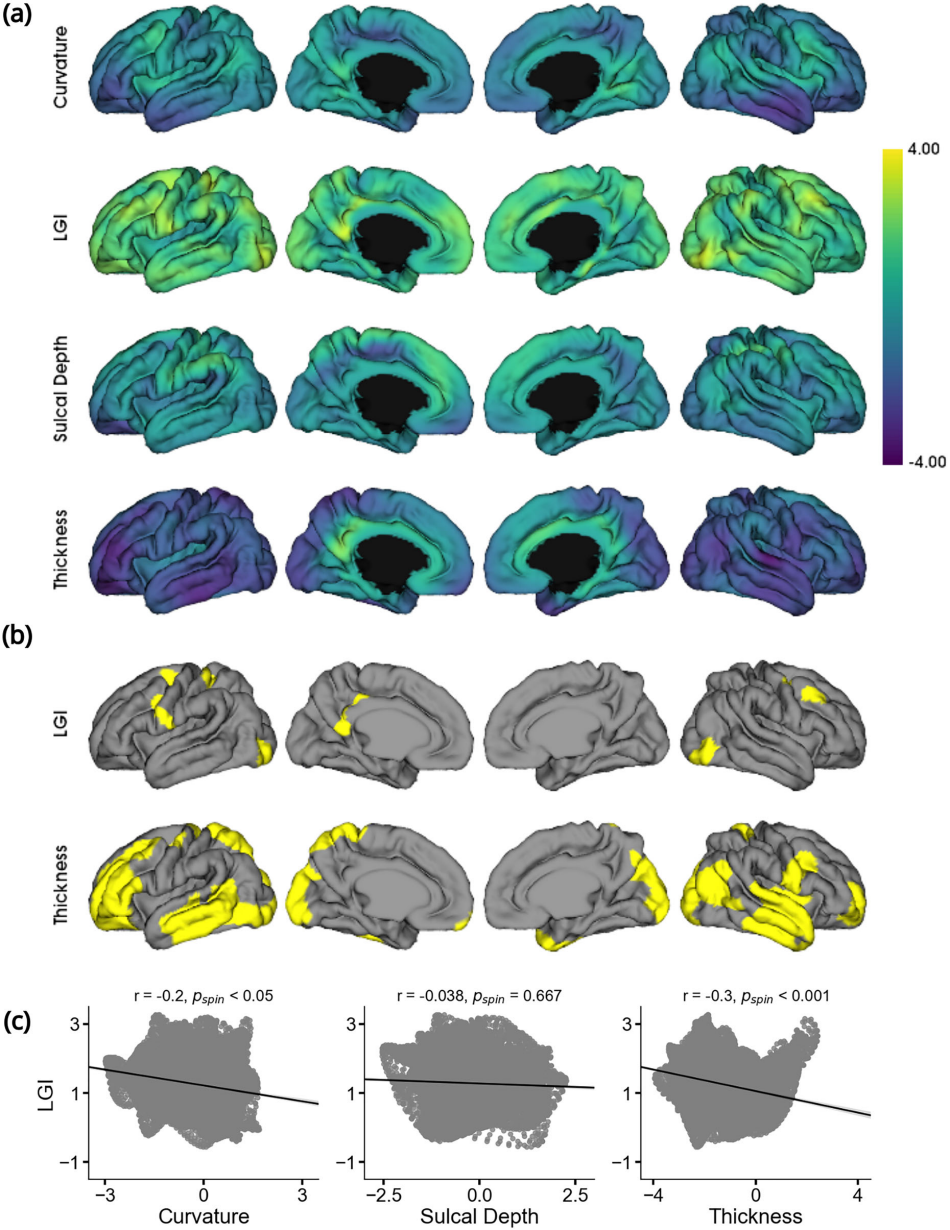
Vertex‐wise longitudinal morphometric analysis. (a) *T*‐maps of group‐wise differences in mean curvature, local gyrification index, sulcal depth, and cortical thickness respectively, computed using vertex‐wise longitudinal mixed‐effects models. Yellow regions indicate larger values in INSVM subjects relative to controls, while blue regions indicate smaller values. (b) Significant clusters after RFT and cluster correction for LGI and cortical thickness. (c) Correlations of the *t*‐map of local gyrification index with those of mean curvature, sulcal depth, and cortical thickness. Significance is assessed using spin tests based on 1000 permutations.

Finally, we conducted a vertex‐wise analysis to assess longitudinal group‐wise morphometric differences between subjects with INSVM and controls. Figure 4 displays the *t*‐maps corresponding to mean curvature, sulcal depth, LGI, and cortical thickness, showing group‐wise differences of subjects with INSVM with respect to controls using the model described in Equation (2), as well as clusters with significant differences in LGI and thickness.

We observed small but positive effects across the entire cortex for curvature and local gyrification index associated with INSVM. Conversely, strong negative effects were found for thickness. Sulcal depth presented more diversified group‐wise differences.

After correction, LGI showed small clusters of significantly higher values in subjects with INSVM compared to controls in various small areas. Cortical thickness, on the other hand, presented significant clusters in the frontal, occipital, and temporal areas, where thickness was considerably lower in subjects with INSVM with respect to healthy controls.

No clusters survived correction for sulcal depth and curvature. After accounting for spatial autocorrelation, a significant negative correlation was found between curvature and LGI (*p* < 0.05), as well as a negative correlation between LGI and thickness (*p <* 0.001).

## 4 Discussion

In this work, we presented a longitudinal neuroimaging analysis of the relationship between ventricular enlargement and cortical development from fetal to the neonatal period. Our study is based on a dataset composed of 30 subjects (15 healthy controls and 15 subjects diagnosed with INSVM), each of which underwent both fetal and neonatal MRI. To establish and quantify the impact of fetal INSVM in cortical development from the gestational to the post‐natal period, we studied ventricular growth in healthy subjects and subjects with INSVM using our longitudinal cohort, performing global, local, and morphometric longitudinal analyses.

Our findings revealed a consistent increase in the volume of the lateral ventricles over time, aligning with previous literature (Ma et al. 2019; Cutler et al. 2020). Neonatal healthy controls showed a ventricular volume of 5.12 ± 0.92, which is within the range of normal pediatric ventricle size at 0‐1 months of age. This corresponds to approximately the 50th percentile as defined by Cutler et al. (2020) for infants aged 0‐3 months and is similar to the mean value for 1‐month‐old infants as reported by Hashimoto et al. (2023).

Subjects with INSVM exhibited significantly larger lateral ventricular volumes compared to controls at both the fetal and neonatal stages (Figure 2). Not only was ventricular volume consistently larger, but a distinct pattern of increase was observed, with subjects with INSVM showing greater expansion of ventricular volume compared to healthy subjects over the gestational period. The ventricular to cortical volume ratio decreased over gestational age for both groups, but the decrease was notably steeper in INSVM subjects, as shown in Supporting Information S5. This phenomenon seems to be compatible with the cortical overgrowth we have observed in our experiments. Despite the initially higher ratio in INSVM subjects, the strong negative trend across both groups indicates that cortical development may follow a relatively typical trajectory in terms of the ventricular to cortical volume ratio, consistent with previous findings Makropoulos et al. (2016), even in the presence of INSVM.

Cortical development in INSVM subjects was characterized using volumetric measurements, cortical thickness, and gyrification descriptors (mean curvature, sulcal depth, and local gyrification index). When comparing global descriptors, our results revealed, for fetal subjects, reduced values for all descriptors in subjects with INSVM compared with controls. For neonatal subjects, we found higher cortical volumes and generally reduced cortical folding in subjects with INSVM compared to healthy controls, as well as reduced gyrification and curvature. Although these findings were non‐significant, they point towards potential differences in cortical structure between INSVM subjects and controls. Interestingly, we found a positive correlation of 0.42 between the global cortical thickness rate of change (i.e., differences between fetal and neonatal scans) and LGI in INSVM subjects (Supporting Information S6), suggesting that increased cortical thickness may be associated with greater gyrification in this group. This correlation was weaker when considering all subjects (*r* = 0.22) and controls alone (*r* = 0.12). These findings imply that altered cortical expansion in INSVM may result in distinct patterns of both cortical thickness and gyrification, possibly reflecting neurodevelopmental changes unique to this condition.

The local volumetric analysis revealed similar results: a general increase of cortical volume in subjects with INSVM compared with healthy controls was observed, but results were only significant in left occipital and right cingulate areas. These findings are in alignment with the literature, as various works showed a relationship between VM and cortical overgrowth alongside specific pathologies such as Soto's syndrome (Palmen et al. 2005; Leventopoulos et al. 2009), hemimegalencephaly (Kalifa et al. 1987), and autism (Palmen et al. 2005). Kyriakopoulou et al. (2014) reported an effective cortical overgrowth in fetuses with VM with respect to healthy controls. In neonates, Gilmore et al. (2008) also showed that subjects with INSVM had significantly larger cortical volumes than control subjects, with no significant differences in absolute white matter volume. Lyall et al. (2012) found that ventricular enlargement persisted at the age of 2 years and was then associated with increases in both gray and white matter volumes. The observed higher cortical volumes may suggest a delayed cortical development trajectory in subjects with INSVM in comparison to their healthy counterparts.

To assess whether there is an effective change in cortical development over time, we conducted a group‐wise longitudinal analysis for several cortical folding descriptors (i.e., local gyrification index, mean curvature, and sulcal depth) and cortical thickness. The morphometric analysis showed a significant reduction in cortical thickness in cortical regions of the frontal, occipital, and parietal lobe, and a higher gyrification index, with differences in cortical thickness being much more prominent. These disparities were not detectable in the global analysis, suggesting that changes caused by INSVM manifest in specific areas of the brain. In the literature, Scott et al. (2013) found significant differences in mean curvature in the parieto‐occipital sulcus in subjects affected by mild VM, whereas Benkarim et al. (2018) reported reduced values of different measures of curvature in the insula, parietal, and occipital lobes in subjects diagnosed with INSVM. Although we found no significant differences in curvature, our results align with the findings present in the literature, showing significant differences in similar brain regions, suggesting that these cortical regions are the most consistently affected in INSVM. Extending this analysis, we found that the effect of LGI had a significantly negative correlation with curvature (which could partly explain the results on curvature found in the literature) and a negative correlation with thickness. The results indicate a growth delay in subjects with INSVM during the late stages of gestation, with thinner cortex compared to normal development (Corbett‐Detig et al. 2011; Clouchoux et al. 2012).

The larger LGI in subjects with INSVM compared to controls is particularly interesting, as we would have expected a reduced folding complexity in subjects with VM, as seen in the literature (Benkarim et al. 2018; Hahner et al. 2019). However, these findings were based on different measures of gyrification and did not directly assess LGI. The results obtained may represent a compensatory mechanism, where the cortex increases its folding to maintain or augment cortical surface area in response to the structural pressure caused by ventricular enlargement. Further investigation is warranted to elucidate the underlying mechanisms driving this increased folding complexity and to determine whether these adaptations are beneficial or potentially maladaptive.

While the lower cortical thickness associated with INSVM subjects shown in for may seem contradictory with the higher cortical thickness and lower LGI shown in 2, those were not significant. Moreover, the model used for the morphometric analysis controls for typical ventricular volume expansion associated with gestational age, meaning that the effect analyzed represents the variation associated specifically with abnormal ventricular change caused by INSVM.

One notable limitation of the present study is the relatively small size of the data set. Ideally, a larger number of subjects would be needed to investigate the differences at each timepoint, especially at the late‐onset stage. Nonetheless, the uniqueness of the collected dataset lies in its multiple MRI acquisitions at the fetal and the neonatal stages, which presents challenges when increasing the subject count. Moreover, perinatal segmentation is subject to challenges related to myelination and the rapid changes in shape and size occurring during this stage of brain development, which make it difficult to accurately segment the different brain tissues and structures using existing methods. As future work, the study could be extended to investigate different volumetric and cortical folding descriptors in larger datasets.

The results of this study show a thinning of the cortex among subjects with INSVM. It remains unclear if INSVM causes these cortical development alterations or if the ventricle enlargement is a product of those alterations. The observed cortical thickness reduction could be caused by the ventricular enlargement, which could exert pressure on the surrounding tissues and lead to a thinner cortex, similar to hydrocephalus (Pisapia et al. 2017). However, given the absence of other detectable structural abnormalities, we cannot rule out that abnormal cortical development could directly play a role in the manifestation of INSVM. However, we lack the data to establish a causal relationship between them.

These results could also be used to study the relationship between cortical folding and neonatal and infant neurobehavioral test scores that assess early cognitive and functional abilities. While the likelihood of adverse neurodevelopmental outcomes is lower in INSVM compared to subjects with severe VM (Ali et al. 2024), there are still associated risks (Sadan et al. 2007; Leitner et al. 2009; Gómez‐Arriaga et al. 2012). Subtle cortical alterations detected through imaging studies might not manifest as overt clinical symptoms initially but could potentially contribute to neurodevelopmental challenges later in life. It remains uncertain whether these patients will eventually suffer negative consequences from VM. Therefore, more longitudinal research is essential to explore the impact of early cortical alterations on neurodevelopmental trajectories and to substantiate any causal links between early brain morphology alterations and later cognitive outcomes.

To conclude, our work examined the relationship between INSVM and specific aspects of cortical development, including changes in gyrification, reduced cortical thickness, and increased overall cortical volume, in a longitudinal cohort of fetuses and neonates. Our analyses showed longitudinally significant changes in cortical development in subjects with INSVM compared to healthy controls. This affectation in cortical development was consistently found across multiple descriptors, resulting in altered cortical folding, cortical volume overgrowth, and reduced cortical thickness in subjects with INSVM. Our findings further suggest that the delay in cortical development was located within the cingulate cortex as well as in regions within the frontal, occipital, and parietal lobes. To the best of our knowledge, this is the first longitudinal MRI study to investigate the impact of INSVM on cortical development in the temporal window spanning the intrauterine and postnatal period. Our work further confirms and consolidates the findings of previous cross‐sectional studies indicating a reduced cortical folding in fetuses with INSVM.

## Author Contributions

**Andrea Urru**: formal analysis, investigation, validation, visualization, writing–original draft. **Oualid Benkarim**: data curation, methodology, software, supervision, validation. **Gerard Martí‐Juan**: formal analysis, investigation, validation, writing–review, and editing. **Nadine Hahner**: conceptualization, data curation, resources, supervision. **Gemma Piella**: funding acquisition, project administration, resources, supervision, writing–review, and editing. **Elisenda Eixarch**: funding acquisition; investigation, project administration; supervision, writing–review, and editing. **Miguel Angel González Ballester**: funding acquisition; investigation; project administration, supervision, writing–review, and editing.

### Peer Review

The peer review history for this article is available at https://publons.com/publon/10.1002/brb3.70255.

## Supporting information



Supporting Information

## Data Availability

Pipeline for data processing is available in https://github.com/urrand/perinatal‐pipeline. Code used for the data analysis is available in https://github.com/urrand/LongitudinalAnalysis. Data is not available publicly; a formal data sharing agreement between Hospital Clínic and the recipient would be needed in order to access the data.

## References

[brb370255-bib-0001] Alexander‐Bloch, A. F. , H. Shou , S. Liu , et al. 2018. “On Testing for Spatial Correspondence Between Maps of Human Brain Structure and Function.” Neuroimage 178: 540–551. 29860082 10.1016/j.neuroimage.2018.05.070 PMC6095687

[brb370255-bib-0002] Ali, F. , F. Gurung , S. Nanda , et al. 2024. “Perinatal and Neurodevelopmental Outcomes of Fetal Isolated Ventriculomegaly: A Systematic Review and Meta‐Analysis.” Translational Pediatrics 13, no. 4: 555–574. 10.21037/tp-23-548. 38715672 PMC11071031

[brb370255-bib-0003] Amlien, I. K. , A. M. Fjell , C. K. Tamnes , et al. 2016. “Organizing Principles of Human Cortical Development—Thickness and Area from 4 to 30 Years: Insights from Comparative Primate Neuroanatomy.” Cerebral Cortex 26, no. 1: 257–267. 25246511 10.1093/cercor/bhu214

[brb370255-bib-0004] Armstrong, E. , A. Schleicher , H. Omran , M. Curtis , and K. Zilles . 1995. “The Ontogeny of Human Gyrification.” Cerebral Cortex 5, no. 1: 56–63. 7719130 10.1093/cercor/5.1.56

[brb370255-bib-0005] Benkarim, O. , G. Piella , I. Rekik , et al. 2020. “A Novel Approach to Multiple Anatomical Shape Analysis: Application to Fetal Ventriculomegaly.” Medical Image Analysis 64: 101750. 32559594 10.1016/j.media.2020.101750 PMC7939052

[brb370255-bib-0006] Benkarim, O. M. , N. Hahner , G. Piella , et al. 2018. “Cortical Folding Alterations in Fetuses With Isolated Non‐Severe Ventriculomegaly.” NeuroImage: Clinical 18: 103–114. 29387528 10.1016/j.nicl.2018.01.006 PMC5790022

[brb370255-bib-0007] Benkarim, O. M. , G. Sanroma , G. Piella , et al. 2018. “Revealing Regional Associations of Cortical Folding Alterations With in Utero Ventricular Dilation Using Joint Spectral embedding.” In: Medical Image Computing and Computer Assisted Intervention—MICCAI 2018, 620–627. Cham, Switzerland: Springer. 10.1007/978-3-030-00931-1_71 PMC6602588 31263804

[brb370255-bib-0008] Benkarim, O. M. , G. Sanroma , V. A. Zimmer , et al. 2017. “Toward the Automatic Quantification of in Utero Brain Development in 3D Structural MRI: A Review.” Human Brain Mapping 38, no. 5: 2772–2787. 10.1002/hbm.23536. 28195417 PMC6867074

[brb370255-bib-0009] Besl, P. , and N. D. McKay . 1992. “A Method for Registration of 3‐D Shapes.” IEEE Transactions on Pattern Analysis and Machine Intelligence 14, no. 2: 239–256.

[brb370255-bib-0010] Brown, T. T. , J. M. Kuperman , Y. Chung , et al. 2012. “Neuroanatomical Assessment of Biological Maturity.” Current Biology 22, no. 18: 1693–1698. 22902750 10.1016/j.cub.2012.07.002 PMC3461087

[brb370255-bib-0011] Chi, J. G. , E. C. Dooling , and F. H. Gilles . 1977. “Gyral Development of the Human Brain.” Annals of Neurology 1, no. 1: 86–93. 560818 10.1002/ana.410010109

[brb370255-bib-0012] Claessens, N. H. , P. Moeskops , A. Buchmann , et al. 2016. “Delayed Cortical Gray Matter Development in Neonates With Severe Congenital Heart Disease.” Pediatric Research 80, no. 5: 668–674. 10.1038/pr.2016.145. 27434120

[brb370255-bib-0013] Clouchoux, C. , D. Kudelski , A. Gholipour , et al. 2012. “Quantitative In Vivo MRI Measurement of Cortical Development in the Fetus.” Brain Structure and Function 217, no. 1: 127–139. 21562906 10.1007/s00429-011-0325-x

[brb370255-bib-0014] Corbett‐Detig, J. , P. A. Habas , J. A. Scott , et al. 2011. “3D Global and Regional Patterns of Human Fetal Subplate Growth Determined in Utero.” Brain Structure and Function 215, no. 3: 255–263. 10.1007/s00429-010-0286-5. 21046152 PMC3041913

[brb370255-bib-0015] Cutler, N. S. , S. Srinivasan , B. L. Aaron , et al. 2020. “Normal Cerebral Ventricular Volume Growth in Childhood.” Journal of Neurosurgery: Pediatrics 26, no. 5: 517–524. 10.3171/2020.5.PEDS20178. 32823266

[brb370255-bib-0016] Cutler, N. S. , S. Srinivasan , B. L. Aaron , et al. 2020. “Normal Cerebral Ventricular Volume Growth in Childhood.” Journal of Neurosurgery: Pediatrics 26, no. 5: 517–524. 32823266 10.3171/2020.5.PEDS20178

[brb370255-bib-0017] Dean, J. M. , E. McClendon , K. Hansen , et al. 2013. “Prenatal Cerebral Ischemia Disrupts MRI‐Defined Cortical Microstructure through Disturbances in Neuronal Arborization.” Science Translational Medicine 5, no. 168: 168ra7. 10.1126/scitranslmed.3004669. PMC3857141 23325800

[brb370255-bib-0018] de Dumast, P. , H. Kebiri , C. Atat , V. Dunet , M. Koob , and M. Bach Cuadra . 2020. “Segmentation of the Cortical Plate in Fetal Brain MRI With a Topological Loss.” *arXiv:2010.12391*.

[brb370255-bib-0019] Ebner, M. , G. Wang , W. Li , et al. 2018. “An Automated Localization, Segmentation and Reconstruction Framework for Fetal Brain MRI.” in: Medical Image Computing and Computer Assisted Intervention—MICCAI 2018, 313–320. Cham, Switzerland: Springer.

[brb370255-bib-0020] Ebner, M. , G. Wang , W. Li , et al. 2019. “An Automated Framework for Localization, Segmentation, and Super‐Resolution Reconstruction of Fetal Brain MRI.” Neuroimage 206: 116324. 31704293 10.1016/j.neuroimage.2019.116324 PMC7103783

[brb370255-bib-0021] Fernández, V. , C. Llinares‐Benadero , and V. Borrell . 2016. “Cerebral Cortex Expansion and Folding: What Have We Learned?” The EMBO Journal 35, no. 10: 1021–1044. 27056680 10.15252/embj.201593701 PMC4868950

[brb370255-bib-0022] Gilmore, J. H. , L. C. Smith , H. M. Wolfe , et al. 2008. “Prenatal Mild Ventriculomegaly Predicts Abnormal Development of the Neonatal Brain.” Biological Psychiatry 64, no. 12: 1069–1076. 18835482 10.1016/j.biopsych.2008.07 PMC2630424

[brb370255-bib-0023] Gómez‐Arriaga, P. , I. Herraiz , J. M. Puente , B. Zamora‐Crespo , N. Núñez Enamorado , and A. Galindo . 2012. “Mid‐Term Neurodevelopmental Outcome in Isolated Mild Ventriculomegaly Diagnosed in Fetal Life.” Fetal Diagnosis and Therapy 31, no. 1: 12–18. 22178749 10.1159/000331408

[brb370255-bib-0024] Griffiths, P. , M. Reeves , J. Morris , et al. 2010. “A Prospective Study of Fetuses With Isolated Ventriculomegaly Investigated by Antenatal Sonography and in Utero MR Imaging.” American Journal of Neuroradiology 31, no. 1: 106–111. 19762458 10.3174/ajnr.A1767 PMC7964094

[brb370255-bib-0025] Habas, P. A. , K. Kim , J. M. Corbett‐Detig , et al. 2010. “A Spatiotemporal Atlas of MR Intensity, Tissue Probability, and Shape of the Fetal Brain With Application to Segmentation.” Neuroimage 53, no. 2: 460–470. 20600970 10.1016/j.neuroimage.2010.06.054 PMC2930902

[brb370255-bib-0026] Hahner, N. , O. Benkarim , M. Aertsen , et al. 2019. “Global and Regional Changes in Cortical Development Assessed by MRI in Fetuses With Isolated Nonsevere Ventriculomegaly Correlate With Neonatal Neurobehavior.” American Journal of Neuroradiology 40, no. 9: 1567–1574. 31467239 10.3174/ajnr.A6165 PMC7048445

[brb370255-bib-0027] Hashimoto, H. , O. Takemoto , K. Nishimoto , G. Moriguchi , M. Nakamura , and Y. Chiba . 2023. “Normal Growth Curve of Choroid Plexus in Children: Implications for Assessing Hydrocephalus due to Choroid Plexus Hyperplasia.” Journal of Neurosurgery: Pediatrics 32, no. 6: 627–637. 10.3171/2023.7.PEDS23218. 37724840

[brb370255-bib-0028] Hayasaka, S. , K. L. Phan , I. Liberzon , K. J. Worsley , and T. E. Nichols . 2004. “Nonstationary Cluster‐Size Inference With Random Field and Permutation Methods.” Neuroimage 22, no. 2: 676–687. 10.1016/j.neuroimage.2004.01.041. 15193596

[brb370255-bib-0029] Huisman, T. A. , A. Tekes , and A. Poretti . 2012. “Brain Malformations and Fetal Ventriculomegaly: What to Look for?.” Journal of Pediatric Neuroradiology 1, no. 3: 185–195.

[brb370255-bib-0030] Jou, R. J. , A. Y. Hardan , and M. S. Keshavan . 2005. “Reduced Cortical Folding in Individuals at High Risk for Schizophrenia: A Pilot Study.” Schizophrenia Research 75, no. 2–3: 309–313. 15885522 10.1016/j.schres.2004.11.008

[brb370255-bib-0031] Kalifa, G. , C. Chiron , N. Sellier , et al. 1987. “HemiMegalencephaly: MR Imaging in Five Children.” Radiology 165, no. 1: 29–33. 3628788 10.1148/radiology.165.1.3628788

[brb370255-bib-0032] King, J. B. , M. P. Lopez‐Larson , and D. A. Yurgelun‐Todd . 2016. “Mean Cortical Curvature Reflects Cytoarchitecture Restructuring in Mild Traumatic Brain Injury.” NeuroImage: Clinical 11: 81–89. 26909332 10.1016/j.nicl.2016.01.003 PMC4735656

[brb370255-bib-0033] Kuklisova‐Murgasova, M. , P. Aljabar , L. Srinivasan , et al. 2011. “A Dynamic 4D Probabilistic Atlas of the Developing Brain.” Neuroimage 54, no. 4: 2750–2763. 20969966 10.1016/j.neuroimage.2010.10.019

[brb370255-bib-0034] Kyriakopoulou, V. , D. Vatansever , S. Elkommos , et al. 2014. “Cortical Overgrowth in Fetuses With Isolated Ventriculomegaly.” Cerebral Cortex 24, no. 8: 2141–2150. 23508710 10.1093/cercor/bht062

[brb370255-bib-0035] Landrieu, P. , B. Husson , D. Pariente , and C. Lacroix . 1998. “MRI‐Neuropathological Correlations in Type 1 Lissencephaly.” Neuroradiology 40, no. 3: 173–176. 9561523 10.1007/s002340050562

[brb370255-bib-0036] Leitner, Y. , O. Stolar , M. Rotstein , et al. 2009. “The Neurocognitive Outcome of Mild Isolated Fetal Ventriculomegaly Verified by Prenatal Magnetic Resonance Imaging.” American Journal of Obstetrics and Gynecology 201, no. 2: e1–6. 10.1016/j.ajog.2009.04.031 19527899

[brb370255-bib-0037] Leventopoulos, G. , S. Kitsiou‐Tzeli , K. Kritikos , et al. 2009. “A Clinical Study of Sotos Syndrome Patients With Review of the Literature.” Pediatric Neurology 40, no. 5: 357–364. 19380072 10.1016/j.pediatrneurol.2008.11.013

[brb370255-bib-0038] Limperopoulos, C. , G. Chilingaryan , N. Sullivan , N. Guizard , R. L. Robertson , and A. J. Du Plessis . 2014. “Injury to the Premature Cerebellum: Outcome is Related to Remote Cortical Development.” Cerebral Cortex 24, no. 3: 728–736. 10.1093/cercor/bhs354. 23146968 PMC3920767

[brb370255-bib-0039] Lockwood Estrin, G. , V. Kyriakopoulou , A. Makropoulos , et al. 2016. “Altered White Matter and Cortical Structure in Neonates With Antenatally Diagnosed Isolated Ventriculomegaly.” NeuroImage: Clinical 11: 139–148. 10.1016/j.nicl.2016.01.012. 26937382 PMC4753810

[brb370255-bib-0040] Lyall, A. E. , S. Woolson , H. M. Wolfe , et al. 2012. “Prenatal Isolated Mild Ventriculomegaly is Associated With Persistent Ventricle Enlargement at Ages 1 and 2.” Early Human Development 88, no. 8: 691–698. 22445211 10.1016/j.earlhumdev.2012.02.003 PMC3386468

[brb370255-bib-0041] Lyu, I. , S. H. Kim , J. B. Girault , J. H. Gilmore , and M. A. Styner . 2018. “A Cortical Shape‐Adaptive Approach to Local Gyrification Index.” Medical Image Analysis 48: 244–258. 29990689 10.1016/j.media.2018.06.009 PMC6167255

[brb370255-bib-0042] Ma, H.‐L. , S.‐X. Zhao , F.‐R. Lv , Z.‐W. Zhang , Y.‐H. Xiao , and B. Sheng . 2019. “Volume Growth Trend and Correlation of Atrial Diameter With Lateral Ventricular Volume in Normal Fetus and Fetus With Ventriculomegaly: A STROBE Compliant Article.” Medicine 98, no. 26:e16118. 31261528 10.1097/MD.0000000000016118 PMC6616102

[brb370255-bib-0043] Makropoulos, A. , P. Aljabar , R. Wright , et al. 2016. “Regional Growth and Atlasing of the Developing Human Brain.” Neuroimage 125: 456–478. 10.1016/j.neuroimage.2015.10.047. 26499811 PMC4692521

[brb370255-bib-0044] Malinger, G. , D. Paladini , K. K. Haratz , A. Monteagudo , G. L. Pilu , and I. E. Timor‐Tritsch . 2020. “Isuog Practice Guidelines (Updated): Sonographic Examination of the Fetal Central Nervous System. Part 1: Performance of Screening Examination and Indications for Targeted Neurosonography.” Ultrasound in Obstetrics and Gynecology: The Official Journal of the International Society of Ultrasound in Obstetrics and Gynecology 56, no. 3: 476–484. 10.1002/uog.22145. 32870591

[brb370255-bib-0045] Melchiorre, K. , A. Bhide , A. Gika , G. Pilu , and A. Papageorghiou . 2009. “Counseling in Isolated Mild Fetal Ventriculomegaly.” Ultrasound in Obstetrics and Gynecology 34, no. 2: 212–224. 19644944 10.1002/uog.7307

[brb370255-bib-0046] Neumane, S. , A. Gondova , Y. Leprince , L. Hertz‐Pannier , T. Arichi , and J. Dubois . 2022. “Early Structural Connectivity Within the Sensorimotor Network: Deviations Related to Prematurity and Association to Neurodevelopmental Outcome.” Frontiers in Neuroscience 16: 932386. 10.3389/fnins.2022.932386. 36507362 PMC9732267

[brb370255-bib-0047] Nordahl, C. W. , D. Dierker , I. Mostafavi , et al. 2007. “Cortical Folding Abnormalities in Autism Revealed by SurfaceBased Morphometry.” Journal of Neuroscience 27, no. 43: 11725–11735. 17959814 10.1523/JNEUROSCI.0777-07.2007 PMC6673212

[brb370255-bib-0048] Palmen, S. J. , H. E. H. Pol , C. Kemner , et al. 2005. “Increased Gray‐Matter Volume in Medication‐Naive High‐Functioning Children With Autism Spectrum Disorder.” Psychological Medicine 35, no. 4: 561–570. 15856726 10.1017/s0033291704003496

[brb370255-bib-0049] Payette, K. , P. de Dumast , H. Kebiri , et al. 2021. “An Automatic Multitissue human Fetal Brain Segmentation Benchmark Using the Fetal Tissue Annotation Dataset.” Scientific Data 8, no. 1: 00. 10.1038/s41597-021-00946-3 PMC8260784 34230489

[brb370255-bib-0050] Payette, K. , R. Kottke , and A. Jakab . 2020. Efficient Multi‐Class Fetal Brain Segmentation in High Resolution MRI Reconstructions With Noisy Labels. arXiv:2009.06275.

[brb370255-bib-0051] Pisapia, J. M. , S. Sinha , D. M. Zarnow , M. P. Johnson , and G. G. Heuer . 2017. “Fetal Ventriculomegaly: Diagnosis, Treatment, and Future Directions.” Child's Nervous System 33, no. 7: 1113–1123. 10.1007/s00381-017-3441-y. 28510072

[brb370255-bib-0052] Raudenbush, S. W. , and A. S. Bryk . 2002. Hierarchical Linear Models: Applications and Data Analysis Methods. Thousand Oaks, California: Sage Publications.

[brb370255-bib-0053] Rutherford, M. 2002. “Magnetic Resonance Imaging of the Brain in Preterm Infants: 24 Weeks' Gestation to Term.” MRI of the Neonatal Brain, 25–49. Philadelphia, Pennsylvania: W B Saunders.

[brb370255-bib-0054] Sadan, S. , G. Malinger , A. Schweiger , D. Lev , and T. Lerman‐Sagie . 2007. “Neuropsychological Outcome of Children With Asymmetric Ventricles or Unilateral Mild Ventriculomegaly Identified in Utero.” BJOG: An International Journal of Obstetrics and Gynaecology 114, no. 5: 596–602. 17439568 10.1111/j.1471-0528.2007.01301.x

[brb370255-bib-0055] Salehi, S. S. , S. R. Hashemi , C. Velasco‐Annis , et al. 2018. Real‐Time Automatic Fetal Brain Extraction in Fetal MRI by Deep Learning. *arXiv:1710.09338*.

[brb370255-bib-0056] Sallet, P. C. , H. Elkis , T. M. Alves , et al. 2003. “Reduced Cortical Folding in Schizophrenia: an MRI Morphometric Study.” American Journal of Psychiatry 160, no. 9: 1606–1613. 12944334 10.1176/appi.ajp.160.9.1606

[brb370255-bib-0057] Salomon, L. , J. Bernard , and Y. Ville . 2007. “Reference Ranges for Fetal Ventricular Width: A Non‐normal Approach.” Ultrasound in Obstetrics and Gynecology 30, no. 1: 61–66. 17506037 10.1002/uog.4026

[brb370255-bib-0058] Scott, J. A. , P. A. Habas , V. Rajagopalan , et al. 2013. “Volumetric and Surface‐based 3D MRI Analyses of Fetal Isolated Mild Ventriculomegaly.” Brain Structure and Function 218, no. 3: 645–655. 22547094 10.1007/s00429-012-0418-1

[brb370255-bib-0059] Serag, A. , P. Aljabar , G. Ball , et al. 2012. “Construction of a Consistent High‐Definition Spatio‐Temporal Atlas of the Developing Brain Using Adaptive Kernel Regression.” Neuroimage 59, no. 3: 2255–2265. 21985910 10.1016/j.neuroimage.2011.09.062

[brb370255-bib-0060] Smith, S. M. 2002. “Fast Robust Automated Brain Extraction.” Human Brain Mapping 17, no. 3: 143–155. 12391568 10.1002/hbm.10062 PMC6871816

[brb370255-bib-0061] Sowell, E. R. , P. M. Thompson , C. M. Leonard , S. E. Welcome , E. Kan , and A. W. Toga . 2004. “Longitudinal Mapping of Cortical Thickness and Brain Growth in Normal Children.” Journal of Neuroscience 24, no. 38: 8223–8231. 15385605 10.1523/JNEUROSCI.1798-04.2004 PMC6729679

[brb370255-bib-0062] Toro, R. , and Y. Burnod . 2005. “A Morphogenetic Model for the Development of Cortical Convolutions.” Cerebral Cortex 15, no. 12: 1900–1913. 15758198 10.1093/cercor/bhi068

[brb370255-bib-0063] Urru, A. , A. Nakaki , O. Benkarim , et al. 2023. “An Automatic Pipeline for Atlas‐Based Fetal and Neonatal Brain Segmentation and Analysis.” Computer Methods and Programs in Biomedicine 230, no. 107334: 1–14. 10.1016/j.cmpb.2023.107334 36682108

[brb370255-bib-0064] Voorhies, W. I. , J. A. Miller , J. K. Yao , S. A. Bunge , and K. S. Weiner . 2021. “Cognitive Insights from Tertiary Sulci in Prefrontal Cortex.” Nature Communications 12, no. 1: 1–14. 10.1038/s41467-021-25162-w PMC8387420 34433806

[brb370255-bib-0065] Wolosin, S. M. , M. E. Richardson , J. G. Hennessey , M. B. Denckla , and S. H. Mostofsky . 2009. “Abnormal Cerebral Cortex Structure in Children With ADHD.” Human Brain Mapping 30, no. 1: 175–184. 17985349 10.1002/hbm.20496 PMC2883170

[brb370255-bib-0066] Yeo, B. T. T. , M. R. Sabuncu , T. Vercauteren , N. Ayache , B. Fischl , and P. Golland . 2010. “Spherical Demons: Fast Diffeomorphic Landmark‐Free Surface Registration.” IEEE Transactions On Medical Imaging 29, no. 3: 650–668. 19709963 10.1109/TMI.2009.2030797 PMC2862393

[brb370255-bib-0067] Yun, H. J. , K. Im , J.‐J. Yang , U. Yoon , and J.‐M. Lee . 2013. “Automated Sulcal Depth Measurement on Cortical Surface Reflecting Geometrical Properties of Sulci.” PLoS ONE 8, no. 2: e55977. 23418488 10.1371/journal.pone.0055977 PMC3572156

