# Supplementary material for: Longitudinal Assessment of Abnormal Cortical Folding in Fetuses and Neonates With Isolated Non‐Severe Ventriculomegaly
Source: Brain Behav. 2025 Jan 20;15(1):e70255. doi: 10.1002/brb3.70255 (PMC11745156; doi:10.1002/brb3.70255)
Supplement: Supplementary file 1 — Supporting Information [file BRB3-15-e70255-s001.pdf]

Supplementary files

1. S1: Additional demographic information

Tables 1 2, 3 summarize the information on ethnicity, education and socioeconomic status of the cohort.

Ethnicity	Caucasian	Latin American	Asian	African
Control	13 (86.67%)	2 (13.33%)	0 (0%)	0 (0%)
VM	12 (80%)	1 (6.67%)	1 (6.67%)	1 (6.67%)

Table 1: Subjects divided by ethnicity, for controls and VM separately. Format is number of subjects (percentage over total %)

Education	Primary education	Secondary education	Superior education
Control	0 (0%)	2 (13.34%)	13 (86.67%)
VM	0 (0%)	5 (33.34%)	10 (66.67%)

Table 2: Subjects divided by education (secondary and superior), for controls and VM separately. Format is number of subjects (percentage over total %)

Status	Employed	Unemployed	Self-employed
Control	15 (100%)	0 (0%)	0 (0%)
VM	8 (53.33%)	6 (40%)	1 (6.67%)

Table 3: Subjects divided by socioeconomic status, for controls and VM separately. Format is number of subjects (percentage over total %)

To evaluate the effects of those variables, we have performed separated general linear models for various key measures, accounting for age, sex and

diagnosis. We did it separately for fetal and neonatal measures. Table 4 show the results.

		Ethnicity	Education	Socioeconomic Status
Fetal	Curvature	0.60 (0.620)	1.12 (0.301)	0.03 (0.969)
	LGI	0.77 (0.523)	1.88 (0.182)	0.14 (0.869)
	Sulcal depth	5.20 (0.007*)	1.32 (0.261)	0.82 (0.451)
	Thickness	0.81 (0.501)	0.75 (0.396)	0.52 (0.604)
	VV	2.50 (0.085)	0.10 (0.752)	1.34 (0.282)
	STV	0.46 (0.710)	0.35 (0.559)	0.50 (0.611)
	CV	0.34 (0.796)	0.18 (0.678)	0.87 (0.430)
Neonatal	Curvature	1.94 (0.151)	1.17 (0.289)	0.03 (0.970)
	LGI	2.36 (0.098)	4.89 (0.036*)	0.88 (0.427)
	Sulcal depth	0.77 (0.522)	2.87 (0.102)	1.32 (0.285)
	Thickness	0.53 (0.668)	0.43 (0.519)	0.52 (0.599)
	VV	2.58 (0.078*)	0.17 (0.683)	0.85 (0.440)
	STV	1.02 (0.402)	0.12 (0.730)	0.26 (0.777)
	CV	1.59 (0.218)	0.00 (0.957)	0.35 (0.711)

Table 4: Results of using generalized linear models, followed by ANOVA analysis, to analize the impact of various demographic variables (columns) for various metrics (rows). Age, sex and diagnosis included as covariates. Results are F-values and corresponding p-values (in parentheses). CV: Cortical volume. VV: ventricle svolume. STV: supratentorial volume. LGI: local gyrification index. *: p<0.05.

	Ethnicity	Education	Socioeconomic status
Curvature	1.59 (0.112)	0.37 (0.715)	0.45 (0.650)
LGI	2.44 (0.015*)	1.41 (0.158)	-0.37 (0.713)
Sulcal depth	-0.25 (0.802)	0.61 (0.541)	-0.08 (0.939)
Thickness	-1.42 (0.157)	-0.71 (0.480)	-0.43 (0.665)
VV	-1.04 (0.300)	0.25 (0.803)	-1.24 (0.215)
STV	1.31 (0.189)	-0.82 (0.411)	0.55 (0.582)
CV	0.77 (0.440)	-0.14 (0.886)	0.02 (0.987)

Table 5: Results of using mixed effect models, to assess the effect of the demographic variables (column), for each of the dependent variables (rows). Age, sex and diagnosis included as covariates. Results are t-values and corresponding p-values (in parentheses). CV: Cortical volume. VV: ventricle svolume. STV: supratentorial volume. LGI: local gyrification index. *: $p<0.05$.

We also have evaluated the relationship between those variables and the measures studied in the study using mixed-effect models while accounting for age, sex and diagnosis. Table 5 show the results from this test.

2. S2: Examples of fetal and neonatal subjects

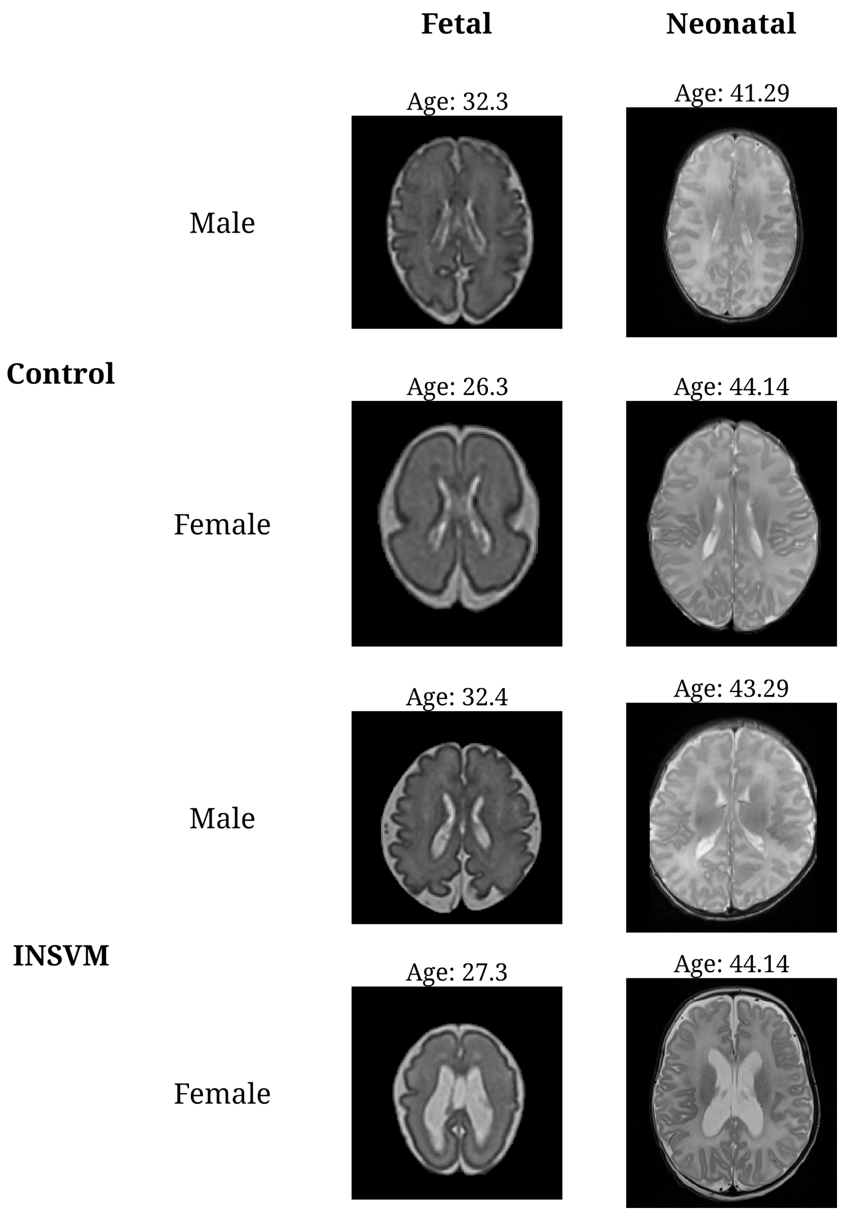

Figure 1: Examples of fetal and neonatal scans at atrial level, for two subjects (Male and female at birth) with different diagnosis, control and INSVM. Age corresponds to the post menstrual age at the time of the scan, in weeks.

3. S3: Mixed effect models results: volumetric analysis

Table 6 shows the results of the mixed effect models for each brain lobe and diagnosis, corrected by age and sex. These results correspond to the graphic in Figure 3 c) from the main text.

Table 6: Regression results by brain lobe for the mixed effect models for each cortical volume region, showing estimates, standard errors, p-values, and confidence intervals for each component. Significant p-values are indicated with asterisks: * $p < 0.05$, ** $p < 0.01$, *** $p < 0.001$.

Lobe	Component	Estimate	SE	**p-value**	CI (95%)
TempLobeL	Age	0.909	0.025	<**0.000*****	[0.860, 0.958]
	Sex (male)	-0.147	0.564	0.794	[-1.253, 0.959]
	Diagnosis (VM)	0.403	0.499	0.419	[-0.574, 1.381]
TempLobeR	Age	0.918	0.029	<**0.000*****	[0.861, 0.974]
	Sex (Male)	-0.199	0.514	0.699	[-1.206, 0.808]
	Diagnosis (VM)	0.393	0.454	0.387	[-0.497, 1.282]
InsulaL	Age	0.114	0.004	<**0.000*****	[0.106, 0.121]
	Sex (Male)	0.014	0.066	0.829	[-0.116, 0.144]
	Diagnosis (VM)	0.042	0.059	0.471	[-0.072, 0.157]
InsulaR	Age	0.120	0.005	<**0.000*****	[0.110, 0.129]
	Sex (Male)	0.070	0.095	0.466	[-0.118, 0.257]
	Diagnosis (VM)	0.076	0.084	0.368	[-0.089, 0.241]
ParietalL	Age	0.764	0.023	<**0.000*****	[0.718, 0.809]
	Sex (Male)	0.133	0.479	0.781	[-0.806, 1.073]
	Diagnosis (VM)	0.368	0.424	0.385	[-0.462, 1.198]
ParietalR	Age	0.751	0.023	<**0.000*****	[0.706, 0.796]
	Sex (Male)	-0.278	0.407	0.494	[-1.076, 0.519]
	Diagnosis (VM)	0.499	0.359	0.165	[-0.205, 1.204]
CingulateL	Age	0.176	0.009	<**0.000*****	[0.158, 0.194]
	Sex (Male)	0.028	0.194	0.886	[-0.352, 0.408]
	Diagnosis (VM)	0.270	0.171	0.115	[-0.066, 0.606]
CingulateR	Age	0.176	0.007	<**0.000*****	[0.162, 0.191]
	Sex (Male)	0.168	0.168	0.317	[-0.161, 0.497]
	Diagnosis (VM)	0.292	0.148	**0.049***	[0.001, 0.583]
FrontalL	Age	1.807	0.056	<**0.000*****	[1.698, 1.916]
	Sex (Male)	-0.193	0.989	0.846	[-2.131, 1.745]
	Diagnosis (VM)	1.599	0.873	0.067	[-0.113, 3.310]
FrontalR	Age	1.825	0.059	<**0.000*****	[1.709, 1.940]
	Sex (Male)	-0.181	1.046	0.862	[-2.232, 1.869]
	Diagnosis (VM)	1.476	0.924	0.110	[-0.335, 3.287]
OccipitalL	Age	1.212	0.035	<**0.000*****	[1.143, 1.281]
	Sex (Male)	-0.099	0.626	0.875	[-1.326, 1.128]
	Diagnosis (VM)	1.112	0.553	**0.044***	[0.028, 2.196]
OccipitalR	Age	1.198	0.036	<**0.000*****	[1.128, 1.268]
	Sex (Male)	-0.357	0.632	0.572	[-1.595, 0.881]
	Diagnosis (VM)	0.893	0.558	0.109	[-0.200, 1.986]

4. S4: Linear regression model for cortical volumes - separated by fetal and neonatal, and on the cortical volume rate of change for each lobe.

Tables 7 and 8 show the results of the linear regression models for each brain lobe and diagnosis, done separately by fetal and neonatal stages, and corrected by gestational age and sex.

Table 7: Fetal regression coefficients (Estimate), standard errors (SE), confidence intervals (CI) and p-values for each brain lobe and diagnosis (VM). Results corrected by gestational age and sex. Significant p-values are indicated with * $p < 0.05$, ** $p < 0.01$, *** $p < 0.001$.

Lobe	Estimate	SE	CI [Lower, Upper]	p-value
TempLobeL	0.535	0.381	[-0.248, 1.318]	0.172
TempLobeR	0.476	0.316	[-0.174, 1.125]	0.144
InsulaL	0.019	0.046	[-0.075, 0.113]	0.679
InsulaR	0.062	0.053	[-0.047, 0.172]	0.253
ParietalL	0.354	0.253	[-0.166, 0.875]	0.173
ParietalR	0.245	0.236	[-0.241, 0.731]	0.310
CingulateL	0.366	0.183	[-0.011, 0.743]	0.056
CingulateR	0.346	0.159	[0.019, 0.674]	**0.039***
FrontalL	0.799	0.466	[-0.159, 1.757]	0.098
FrontalR	0.847	0.496	[-0.174, 1.867]	0.100
OccipitalL	0.425	0.348	[-0.290, 1.140]	0.233
OccipitalR	0.277	0.333	[-0.407, 0.960]	0.413

Table 8: Neonatal regression coefficients (Estimate), standard errors (SE), confidence intervals (CI) and p-values for each brain lobe and diagnosis (VM). Results corrected by gestational age and sex. Significant p-values are indicated with * $p < 0.05$, ** $p < 0.01$, *** $p < 0.001$.

**Lobe**	**Estimate**	**SE**	**CI [Lower, Upper]**	**p-value**
TempLobeL	-0.016	0.796	[-1.652, 1.620]	0.984
TempLobeR	-0.010	0.835	[-1.726, 1.706]	0.991
InsulaL	-0.001	0.093	[-0.191, 0.190]	0.994
InsulaR	-0.002	0.122	[-0.254, 0.249]	0.985
ParietalL	-0.032	0.680	[-1.430, 1.366]	0.963
ParietalR	0.469	0.664	[-0.896, 1.833]	0.486
CingulateL	0.020	0.239	[-0.472, 0.512]	0.933
CingulateR	0.104	0.194	[-0.294, 0.503]	0.596
FrontalL	1.317	1.417	[-1.595, 4.230]	0.361
FrontalR	0.913	1.450	[-2.067, 3.894]	0.534
OccipitalL	1.035	0.844	[-0.700, 2.770]	0.231
OccipitalR	0.689	0.864	[-1.088, 2.465]	0.433

Table 9 shows the results of the linear model between the rate of change of the cortical volume between fetal and neonatal and diagnosis, corrected by gestational age difference between fetal and neonatal acquisitions, and sex.

Table 9: Regression regression coefficients (Estimate), standard errors (SE), confidence intervals (CI) and p-values for each brain lobe and diagnosis (VM). Results corrected by difference in gestational age and sex. Significant p-values are indicated with * $p < 0.05$, ** $p < 0.01$, *** $p < 0.001$.

**Lobe**	**Estimate**	**SE**	**CI [Lower, Upper]**	**p-value**
TempLobeL	-0.008	0.029	[-0.067, 0.051]	0.786
TempLobeR	-0.010	0.029	[-0.070, 0.050]	0.739
InsulaL	0.009	0.027	[-0.046, 0.064]	0.745
InsulaR	-0.020	0.026	[-0.074, 0.034]	0.463
ParietalL	-0.006	0.029	[-0.064, 0.053]	0.841
ParietalR	0.014	0.028	[-0.043, 0.071]	0.626
CingulateL	-0.098	0.046	[-0.192, -0.003]	**0.043***
CingulateR	-0.077	0.041	[-0.161, 0.007]	0.071
FrontalL	0.000	0.020	[-0.041, 0.041]	0.984
FrontalR	-0.009	0.021	[-0.052, 0.034]	0.664
OccipitalL	0.004	0.021	[-0.040, 0.048]	0.854
OccipitalR	0.008	0.022	[-0.038, 0.054]	0.725

5. S5: Correlation between LGI and gestational age with ratio between ventricle volume and cortical volume

Figure 2 shows the correlation between LGI and gestational age with the ratio between ventricule volume and cortical volume. The correlation coefficient and the linear regression line are shown for each plot. We first show the correlation separated by diagnosis, and then combined.

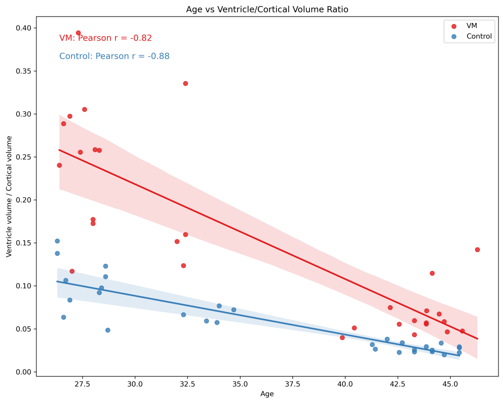

(a) Ventricle volume/Cortical volume vs Age, separated by diagnosis.

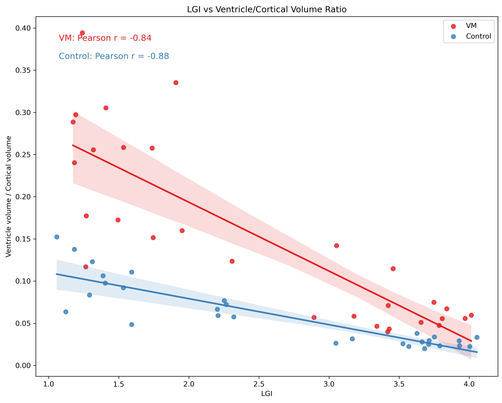

(b) Ventricle volume/Cortical volume vs LGI, separated by diagnosis.

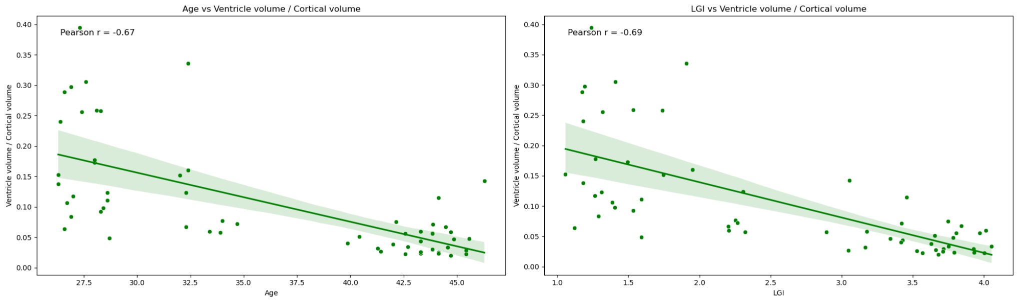

(c) Correlation between Ventricle volume/Cortical volume ratio and LGI (left), Age (right). All subjects and timepoints.

Figure 2: Correlation between Ventricle volume/Cortical volume ratio and Age (top left), LGI (top right), separated by diagnosis and timepoint, and with all subjects (bottom). Each subplot shows a Pearson correlation coefficient with the corresponding linear regression line and confidence interval.

6. S6: Correlation between LGI and changes in cortical thickness

Figure 3 shows the correlation between LGI and the rate of change in total cortical thickness. The correlation coefficient and the linear regression line are shown for each plot.

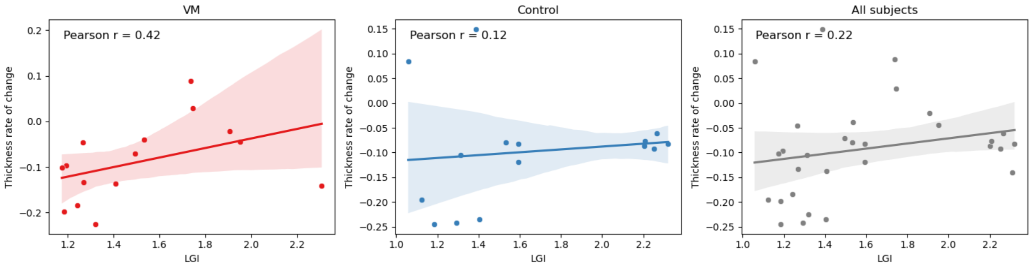

Figure 3: Correlation between Local Gyrification Index (LGI) and cortical thickness rate of change across three groups: VM (ventriculomegaly), control, and all subjects. Pearson correlation coefficients (r) are reported for each group. Shaded regions represent the 95% confidence intervals.
